# Congestive ischemic colitis occurring after resection of left colon cancer: 4 case series

**DOI:** 10.1186/s40792-020-00919-5

**Published:** 2020-07-20

**Authors:** Takatsugu Fujii, Shigeo Toda, Yuki Nishihara, Yusuke Maeda, Kosuke Hiramatsu, Yutaka Hanaoka, Rikiya Sato, Shuichiro Matoba, Masashi Ueno, Hiroya Kuroyanagi

**Affiliations:** Department of Gastrointestinal Surgery, Toranomon Hospital, 2-2-2 Toranomon, Minato-ku, Tokyo, 105-8470 Japan

**Keywords:** Ischemic colitis, Colon cancer, Anastomosis, Colectomy

## Abstract

**Background:**

Ischemic colitis can occur after colectomy and is sometimes difficult to treat. We report 4 cases of refractory, delayed onset, regional congestive colitis occurring on the anal side of the anastomosis after laparoscopic left hemicolectomy.

**Case presentation:**

A total of 191 patients underwent surgery for left colon cancer (transverse, descending, and sigmoid colon cancer) at our hospital from January 2012 to December 2017. During the procedures, the left colic artery (LCA) or sigmoid colic artery (SA) was dissected, the superior rectal artery (SRA) was preserved, and the inferior mesenteric vein (IMV) was dissected at the inferior margin of the pancreas. Congestive ischemic colitis due to venous return dysfunction occurred in 4 cases (2.1%), 5 to 34 months postoperatively. The patients had diarrhea and blood in the stool. On computed tomography (CT), the patients exhibited continuous intestinal edema and high-density adipose tissue from the anastomosis site to the rectum. Contrast enhancement showed dilation of the vasa recti and arteries from the inferior mesenteric artery (IMA) to the SRA. Three patients improved with long-term intestinal rest; in 1 case, the stenosis did not improve and required colorectal resection.

**Conclusion:**

Diagnoses were easy in these cases, but treatment was prolonged and surgery was necessary in 1 case. While this condition is rare, caution is warranted as it is difficult to treat.

## Introduction

Ischemic colitis is a condition involving mucosal inflammation and ulceration due to reduced blood flow to the colonic mucosa. The ischemia is caused by vascular factors such as arteriosclerosis, factors on the intestinal side that affect the intestine due to increased intestinal pressure from constipation or other reasons, and factors associated with congestion due to venous return dysfunction [1, 2].

How best to treat and preserve blood vessels during surgery for left side colon cancer is still a topic of debate. The appropriateness of lymph node dissection and how to achieve a balance with postoperative complications such as anastomotic leakage and ischemic colitis are also still under debate [3–5].

Here, we report our experience with 4 cases of congestive ischemic colitis that occurred after left side colon cancer surgery. We believe the colitis was caused by preserving the area from the inferior mesenteric artery (IMA) to the superior rectal artery (SRA), resecting the left colic artery (LCA) or sigmoid colic artery (SA), and dissecting the inferior mesenteric vein (IMV) both at the inferior margin of the pancreas and at the root of LCA or SA level. Several months after the procedures, the patients developed continuous ischemic colitis from the anastomosis site to the rectum with dilation of the vasa recti and arteries, as seen on computed tomography (CT).

We describe the pathophysiology, clinical presentations, and incidence rate of 4 cases of postoperative congestive colitis (PCC).

## Case presentation

### Case 1: a 75-year-old woman (Table 1)

Laparoscopic left hemicolectomy was performed for transverse colon cancer. The LCA was resected; the SA and SRA were preserved. The IMV is dissected both at the inferior margin of the pancreas and at the root of LCA level. Functional end-to-end anastomosis was performed. Nineteen months postoperatively, the patient developed diarrhea and bloody stool. CT revealed continuous edematous changes from the anastomosis site to the rectum and a high-density adipose tissue from the anastomosis site to the rectum. The patient was made nothing per os and placed under intravenous hyperalimentation management; her symptoms improved and oral intake was restarted 3 months after admission.

**Table 1:** Details of the operative procedures

Case	Age(Years)/Gender	Past medical history	Ligated artery	Proximal/ Distal Margin(cm)	Distance between the sacral promotory and anastomotic site(cm)	Operative time(min)	Blood loss(ml)	Anastomosis	f-stage
1	75/F	Appendicitis	LCA	2.4/12.3	11	312	0	FEEA	II
2	57/M	Hypertension	LCA,SA1	10.1/7.4	2.5	268	0	DST	I
3	67/M	Diabetes mellitus, Hyperlipidemia	LCA,SA1,SA2	5/15.9	2	315	0	DST	I
4	60/M	cholecectomy	LCA	8.2/8.3	7.5	241	0	FEEA	II

*LCA* left colic artery, *SA* sigmoid artery, *FEEA* functional end-to-end anastomosis, *DST* double stapling technique

### Cases 2 and 3: 57- and 67-year-old men

**Fig. 1:**
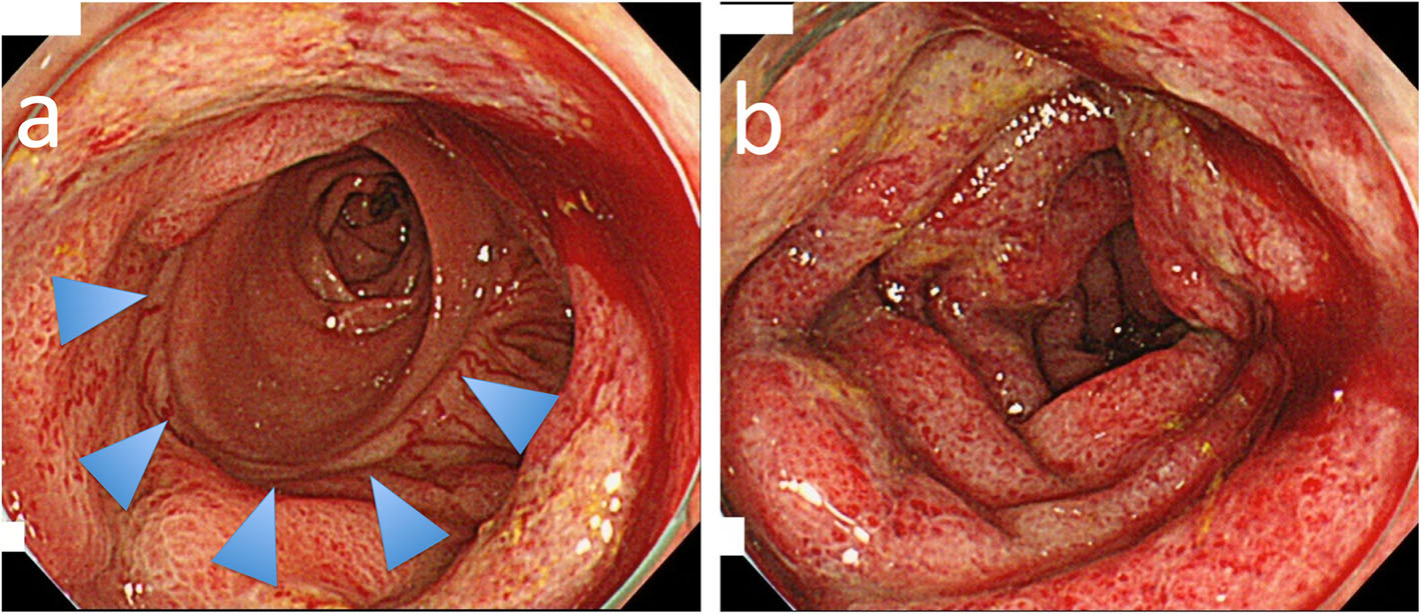
**a** The oral side of the anastomosis was normal colon mucosa. The arrow indicates anastomosis. **b** The anal side of anastomosis was full circumferential inflammation continued to the rectum

They developed colitis 6 and 34 months after surgery. The LCA was resected; the SA and SRA were preserved. The IMV is dissected both at the inferior margin of the pancreas and at the root of SA level. Case 2 colonoscopy revealed the oral side of the anastomosis was normal colon mucosa (Fig. 1a) and the anal side of anastomosis was full circumferential inflammation continued to the rectum (Fig. 1b).

They were hospitalized and were made nothing per os. Intravenous prostaglandin E1 (PGE1) was administered but showed no improvement. They clinically improved and restarted oral intake. The length of hospital stay was 55 and 52 days.

### Case 4: a 60-year-old man (Table 2)

**Fig 2:**
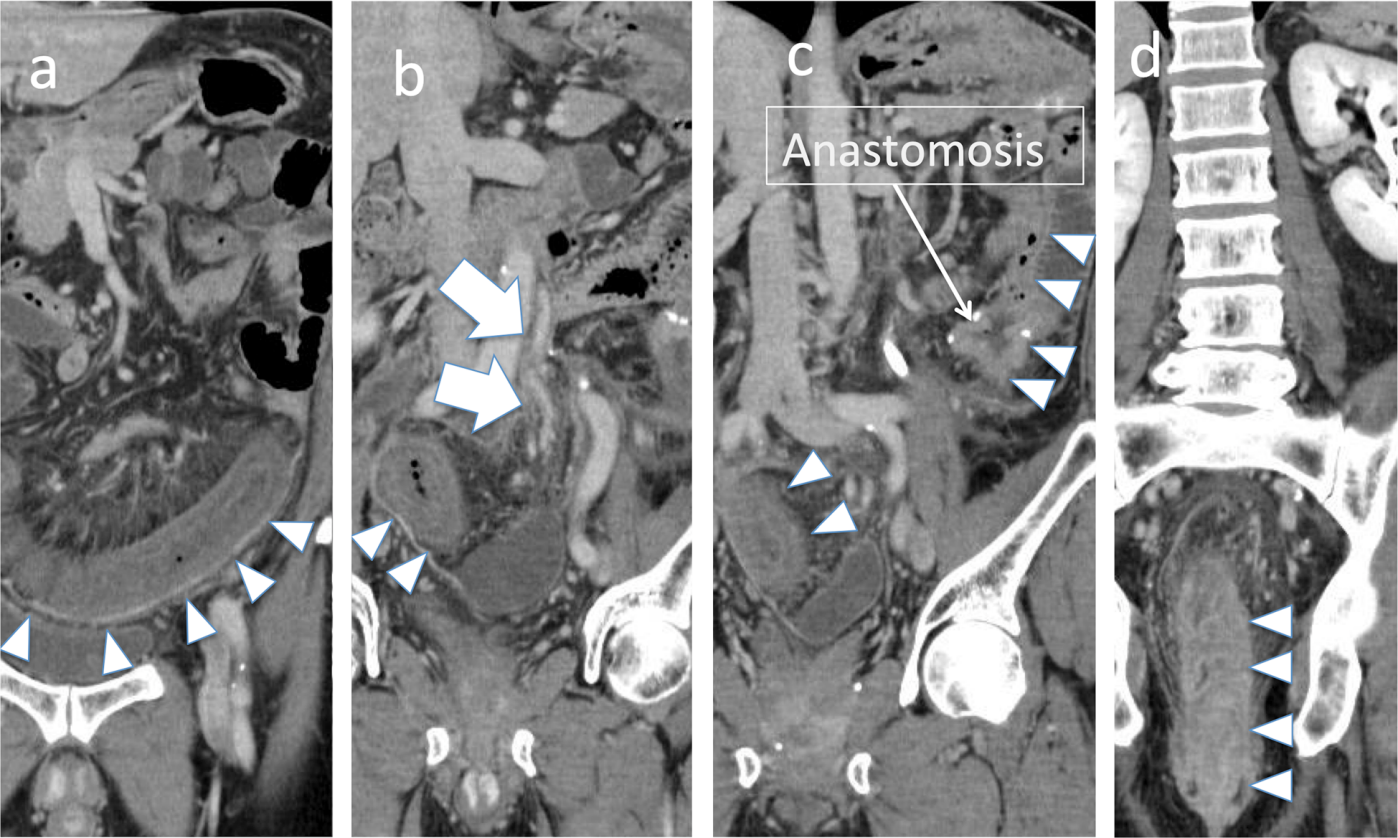
**a** There are continuous edematous changes from the anastomosis site to the rectum (triangular arrow). **b** Contrast enhancement shows the IMA (arrow). **c**, **d** The oral side of the intestines to the anastomosis seems to be normal. IMV, inferior mesenteric vein

Laparoscopic left hemicolectomy was performed for descending colon cancer. The LCA and SA were resected, the SRA was preserved, and the IMV was ligated both at the inferior margin of the pancreas and at the root of SA level. The patient was discharged from the hospital on postoperative day 7. Five months postoperatively, the patient developed diarrhea and abdominal pain, followed by blood in the stool and a fever of approximately 38 °C. A blood test showed a white blood cell (WBC) count of 18,200/μL and C-reactive protein level of 13.6 mg/μL. CT indicated continuous intestinal edema from the anastomosis site to the rectum and signs of increased panniculitis in the surrounding area. Contrast enhancement from the IMA to the SA and SRA showed slight dilation of the straight veins and arteries in the area of intestinal edema (Fig. 2). Colonoscopy showed stenosis 15 cm from the anal verge, with mucosal shedding and bleeding on the oral side. The patient was made nothing per os and placed under intravenous hyperalimentation management. Intravenous PGE1 and antibiotics (cefmetazole, metronidazole) were administered but no improvement was observed. On hospital day 31, temporary ileostomy was performed. However, no improvement in the stenosis was observed on colonoscopy even after 3 months had passed. Laparoscopic low anterior resection was performed to remove the intestinal stenosis, including the anastomosis site.

**Table 2:** Details of the treatment received by the 4 cases

Case	Time to onset from the initial surgery(month)	WBC/CRP	Symptoms	Hospital stay (days)	Treatment
1	19m	8600/1.2	diarrhea,bloody stool	157	CMZ
2	6m	13400/2.2	diarrhea	55	CMZ,PGE
3	34m	12400/2.2	diarrhea	52	PGE
4	5m	18200/13.6	diarrhea,abdominal pain,fever	84※	PGE,CMZ,MTZ, Ileostomy,low anterior resection

*CMZ* cefmetazole, *PGE* prostaglandin, *MTZ* metronidazole

*The patient underwent ileostomy and was temporarily discharged after 56 days in the hospital. He was readmitted 108 days later to undergo laparoscopic low anterior resection. He was discharged from the hospital after 28 days

Pathological examination showed complete stenosis of the colon but no necrosis, suggesting ischemia due to chronic and incomplete stenosis. There were no signs of calcification or atheroma in the arteries, though mild dilation of the arteries and veins with surrounding fibrosis was observed. No metastasis or recurrence was observed.

## Discussion

Among the cases of ischemic colitis that occurred after left side colectomy, we treated 4 cases (2.1%) in which the congestion that caused ischemic colitis occurred because the arterial branch (SRA) was preserved and the IMV was resected near the root.

Among the surgeries for colon cancer performed at our hospital from January 2012 to December 2017, left side colon cancer surgery (transverse to sigmoid colon) was performed in 753 patients. These included 191 cases of left colectomy in which the SRA was preserved and the LCA or SA was resected; the IMV was dissected both at the inferior margin of the pancreas. PCC occurred in 4 of these patients (2.1%). PCC is defined as continuous edematous changes from the anastomosis site to the rectum, high-density adipose tissue around the mesocolon, and dilation of the vasa recti and arteries as seen on CT, as well as signs of ischemic colitis on colonoscopy.

All cases required long-term hospitalization for treatment, and in 1 case, the stenosis needed to be removed because it did not improve.

Ischemic colitis has been reported in 0.83% of cases when the IMA was treated at the root during resection of sigmoid colon or rectal cancer [3], and this condition is known to be associated with suture failure and prognosis [4, 6]. Most cases of postoperative ischemic colitis are associated with reduced arterial blood flow. However, in a previously reported case in which the blood vessels were treated similarly to our cases, ischemic colitis on the anal side of the anastomosis that occurred 1 year postoperatively may have been PCC [7].

**Fig. 3:**
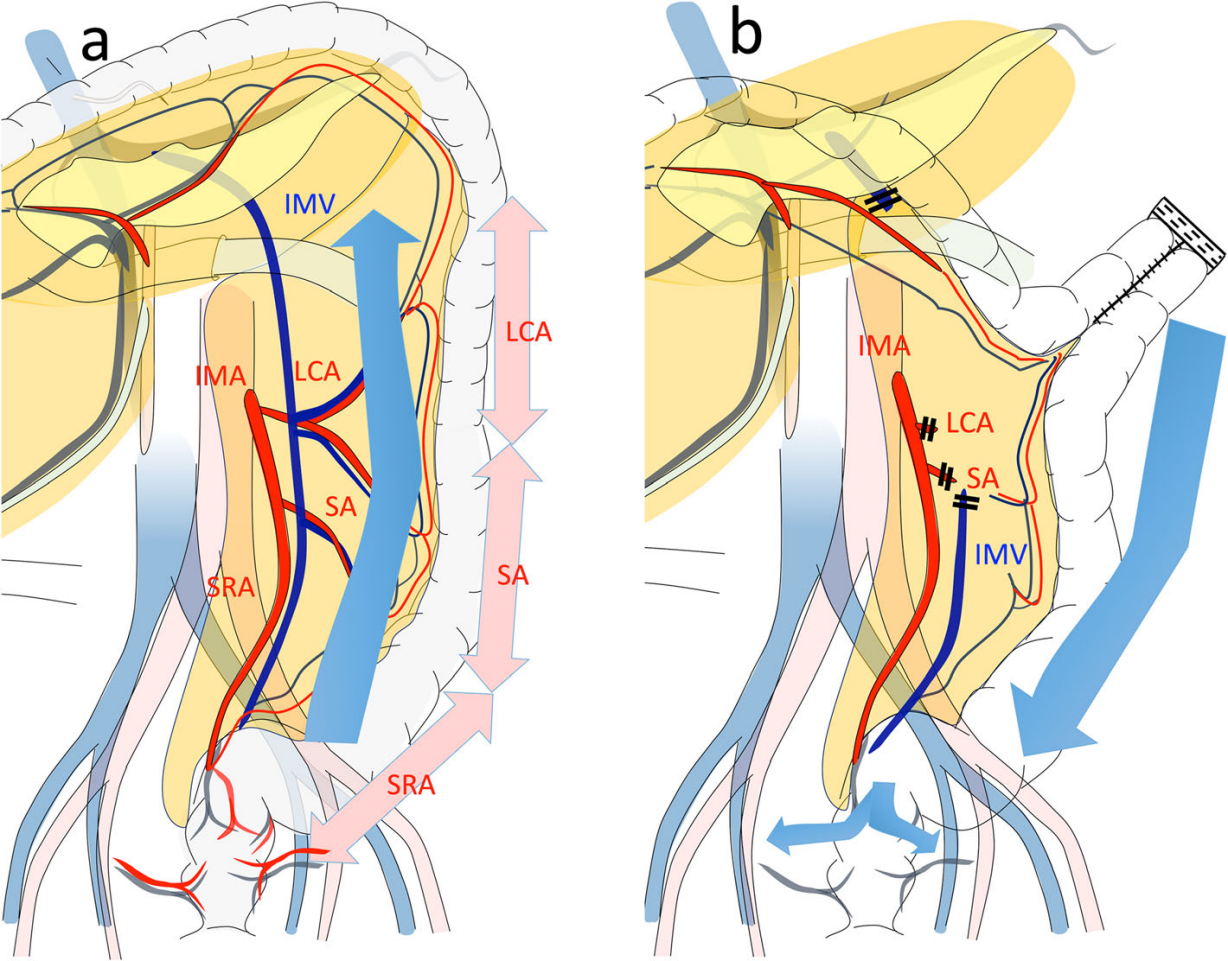
**a** The IMV returns from the left transverse colon to the rectum. **b** When the SRA and SA are preserved and the IMV is dissected both at the inferior margin of the pancreas and at the root of SA (cases 2–4) level, venous blood from the anastomosis to the rectum mainly returns via the rectal vein. In case 1, the distal branches of IMV dissected at the root of LCA level

The arterial blood flow to the left colon comes from the IMA, which generally branches into the LCA, SA, and SRA (Fig. 3a). In contrast, venous blood in the left colon returns to the descending colon, sigmoid colon, and rectum via the IMV. In left colon cancer, selecting which arteries to preserve and which to remove depends on the stage and location of the tumor. When the arterial branch is preserved, treating the IMV near the root may lead to imbalances in the venous return, which can create congestion (Fig. 3b). In the present cases, contrast enhancement from the IMA to the marginal artery and vasa recti indicated that ischemia due to arterial obstruction was unlikely (Fig. 2). We certainly confirmed that the marginal artery and the vein around the anastomosis were preserved. If there had been the damage of vessel or bleeding, the congestive ischemic colitis or even anastomotic leakage could have happened earlier.

Generally, occlusion of the IMV is idiopathic, due to deficiency of protein S or C, or accompanies coagulation abnormalities due to antiphospholipid antibody syndrome and presents with symptoms similar to PCC, such as mesenteric panniculitis of the colon or colitis [8–10]. Further, venous occlusion has been reported in a case of idiopathic mesenteric panniculitis [11]. In the present cases, because the IMV was resected at the inferior margin of the pancreas, we believe that venous return insufficiency caused the congestion and mesenteric panniculitis. The cause of imbalance was not clear, as mesenteric inflammation or microvascular mesenteric vein thrombosis caused venous occlusion and it may induce imbalance of blood flow.

PCC has the following 3 clinical features. First, congestion of venous return in the IMV region causes congestive colitis continuously from the anastomosis site to near the rectum. Venous return may be reduced starting from the anastomosis site when the residual sigmoid colon (return region) is long. The distance between the sacral promontory and the anastomotic site of cases 1 and 4 was longer than cases 2 and 3. Cases 1 and 4 needed more time to treatment (Table 1). In addition, continuous edematous changes from the anastomosis site to the rectum in CT are characteristic and make diagnosis easy.

Second, because it is venous congestion, the condition does not develop immediately after surgery; in the present cases, the condition took a median of 12.5 months (5–34 months) to appear (Table 1).

Third, all 4 cases experienced repeated watery stool from 1 week to 1 month before hospitalization. This was followed by symptoms such as abdominal pain and fever, after which diagnosis was made using CT.

Regarding treatment, resting the intestinal tract was the most effective. However, in case 4, the inflammation was severe and the stenosis on the anal side of the anastomosis did not improve even after ileostomy, making resection of the portion with colitis necessary. The inflammatory reactions did not improve even after administering antibiotics. While PEG1 has been reported to be effective for improving blood flow in postoperative ischemic colitis [12], it had no clear effects in cases 2, 3, and 4 (Table 2).

Trying to preserve as much of the IMV as possible could help prevent venous congestion, but preserving the main IMV trunk while resecting the branches is technically difficult. In transverse colon cancer, the area around the IMV is within the dissection range, which means this strategy could impact recurrence. When the IMV needs to be treated at the inferior margin of the pancreas for oncological reasons, the IMA to the SRA should not be preserved, the IMA should be dissected at the root, and anastomosis should be performed at a site with good blood flow. Alternatively, prevention may be possible by ensuring enough of the intestine on the anal side of the anastomosis is resected.

## Conclusion

Our experience with 4 cases of PCC showed that while diagnosis is easy, treatment can be prolonged and there is a lack of effective treatments. Three patients improved with a conservative approach and did not experience recurrence, but 1 patient needed surgical resection due to irreversible stenosis. PCC is a rare condition and more cases need to be examined to understand its pathophysiology and how to prevent it.

## Data Availability

The datasets supporting the conclusions of this article are included within the article.

## Abbreviations

IMA: Inferior mesenteric artery
LCA: The left colic artery
SA: Sigmoid colic
SRA: Superior rectal artery
IMV: Inferior mesenteric vein
WBC: White blood cell
CT: Computed tomography
PCC: Postoperative congestive colitis
PGE1: Prostaglandin E1
